# Supplementary material for: A UMLS-based spell checker for natural language processing in vaccine safety
Source: BMC Med Inform Decis Mak. 2007 Feb 12;7:3. doi: 10.1186/1472-6947-7-3 (PMC1805499; doi:10.1186/1472-6947-7-3)
Supplement: Additional file 13 — RAP application directory. Sets up directory for RAP (RDF application for PHP) source code [file 1472-6947-7-3-S13.gz › rap/doc/phpdoc/rdql/RdqlMemEngine.html]

xml version="1.0" encoding="iso-8859-1"?


Docs For Class RdqlMemEngine


## Class RdqlMemEngine

Description

Description |
Methods (details)

This class performes as RDQL query on a MemModel.

Provided an rdql query parsed into an array of php variables and constraints at first the engine searches for tuples matching all patterns from the WHERE clause of the given RDQL query. Then the query result set is filtered with evaluated boolean expressions from the AND clause of the given RDQL query.

History:

- access: public
- author: Radoslaw Oldakowski <radol@gmx.de>
- version: V0.9.1

Located in /rdql/RdqlMemEngine.php (line 36)

```
Object
   |
   --RdqlEngine
      |
      --RdqlMemEngine
```

Method Summary

Description |
Methods (details)

array
&queryModel
(object MemModel &$memModel, array &$parsedQuery, [boolean $returnNodes = TRUE])

Methods

Description |
Methods (details)

queryModel (line 79)

Perform an RDQL Query on the given MemModel.

- return: [][?VARNAME] = object Node (if $returnNodes = TRUE) OR array [][?VARNAME] = string
- access: public

array

&queryModel
(object MemModel &$memModel, array &$parsedQuery, [boolean $returnNodes = TRUE])

- object MemModel
  &$memModel
- array
  &$parsedQuery: (the same format as $this->parsedQuery)
- boolean
  $returnNodes

#### Inherited Methods


Inherited From Object

> Object::toString()

Documentation generated on Thu, 7 Jul 2005 13:43:16 +0200 by phpDocumentor 1.3.0RC3
